# Influence of Restorative Material on the Distribution of Loads to the Bone in Hybrid Abutment Crowns—In Vitro Study

**DOI:** 10.3390/medicina59071188

**Published:** 2023-06-22

**Authors:** Rafael Garcia Martins, Tayna Silva de Castro, Luciano Lauria Dib, Sergio Alexandre Gehrke, Alfredo Mikail Melo Mesquita

**Affiliations:** 1 Department of Implantology, Paulista University—UNIP, São Paulo 04026-002, Brazil; rafael@diventa.com.br (R.G.M.); taynasc@gmail.com (T.S.d.C.); luciano.dib@docente.unip.br (L.L.D.); 2 Department of Biotechnology, Universidad Católica de Murcia (UCAM), 30107 Murcia, Spain; sergio.gehrke@hotmail.com

**Keywords:** stress dissipation, dental implant, design/computer-aided manufacturing, ceramic materials

## Abstract

***Background***: The objective of this study was to evaluate the load transmitted to the peri-implant bone by seven different restorative materials in single-unit rehabilitations with morse taper implants using a strain gauge. ***Materials***: In a polyurethane block that simulated type III bone, a morse taper platform implant was installed (3.5 × 11 mm) in the center and 1 mm below the test base surface, and four strain gauges were installed around the implant, simulating the mesial, distal, buccal and lingual positions. Seven similar hybrid abutment crowns were crafted to simulate a lower premolar using different materials: 1—PMMA; 2—glass ceramic over resin matrix; 3—PEEK + lithium disilicate; 4—metal–ceramic; 5—lithium disilicate; 6—zirconia + feldspathic; 7—monolithic zirconia. All groups underwent axial and oblique loads (45 degrees) of 150 N from a universal testing machine. Five measurements (*n* = 5) were performed with each material and for each load type; the microdeformation data underwent statistical analysis. The data were obtained in microdeformation (με), and the significance level was set at *p* ≤ 0.05. ***Results***: There was no statistically significant difference in the evaluation among the materials under either the axial load or the oblique load at 45 degrees. In turn, in the comparison between axial load and oblique load, there was a difference in load for all materials. ***Conclusion***: The restorative material did not influence the load transmitted to the bone. Furthermore, the load transmitted to the bone was greater when it occurred obliquely at 45° regardless of the material used. In conclusion, it appeared that the different elastic modulus of each material did not influence the load transmission to the peri-implant bone.

## 1. Introduction

In natural teeth, the periodontium transmits the forces applied to the teeth along the entire root surface to the bone [1]. On the other hand, in implant-supported prostheses, the masticatory loads are transmitted directly to the bone and are concentrated on the crest of the ridge [2]. It is suggested that the absence of periodontal ligaments around the implants decreases proprioception and shock absorption, and the consequent low movement may be a factor that indicates that implants are less tolerant to occlusal loads than natural teeth and are subject to complications related to ceramic fracture and the chipping of implant crowns and bridges [3].

In this sense, studies using different methodologies have suggested that occlusal loads are concentrated in the peri-implant bone and that excessive stress can lead to bone resorption depending on bone quality [4]. In this regard, a literature review evaluating seven clinical human studies with a minimum of 10 implants installed evaluated clinical and radiographic parameters relevant to the diagnosis of occlusal overload of oral implants and suggested an association between occlusal overload and peri-implant bone loss [5].

In a recent literature review, we sought to answer the following question: Can traumatic occlusal forces lead to peri-implant bone loss? Based on animal models, the conclusion was that the literature suggests there may be a relationship between peri-implant bone loss and occlusal overload when a pathological load is applied. In humans, the results are controversial, and the evidence is too limited to support a cause–effect relationship [6].

Furthermore, it is also known that if the load transferred to an implant or peri-implant bone exceeds physiological limits, there may be failure in rehabilitation or even loss of osseointegration [7,8]. Therefore, any factors that may reduce the stress generated in the implant/prosthesis system must be regarded and studied.

The load transfer at the bone–implant interface depends on, among other factors, the type of load, quantity and quality of bone tissue, implant geometry, position, number and linear arrangement of implants, occlusal surface size, prosthetic interfaces, parafunctional occlusal habits, bite force, primary mechanical stability and type of prosthetic retention. Thus, it appears that when the occlusal force exceeds the stress absorbance capacity of the osseointegrated interface, the implant may fail [7,9].

Biomechanical aspects play a fundamental role in the in vivo behavior of osseointegrated implants [10]. It has been reported that excessive maxillary bone stress or deformations can affect the peri-implant marginal bone, as well as decrease peri-implant bone density [11]. According to some studies, occlusal overload is one of the factors contributing to bone loss around dental implants [12].

The biomechanical behavior of implant-supported prostheses and implants can be analyzed through three different methods: (1) finite elements; (2) photoelastic analysis; and (3) strain gauge analysis [13].

In a recent study, Datte et al. also evaluated the use of different restorative materials in another methodology (finite element analysis) and concluded that the restorative materials used in the manufacture of monolithic crowns on unitary morse taper implants are not capable of influencing bone strain [14]. In a recent photoelastic analysis evaluating the load transmission to the bone of different restorative materials, it was demonstrated that the load transmission is influenced by the materials, of which zirconia and lithium disilicate presented the highest stress [15].

Strain gauge analysis makes it possible to obtain numerical data on the force acting on the implant and transferred to the peri-implant tissues [13].

In turn, it is a strain measurement technique that can be used to measure variations in pressure, temperature and, in this case, volumetric deformation. It can be applied to the most diverse activities and covers different areas of engineering and science. This technique allows real data to be obtained on the force exerted on the implant crowns and transferred to the support structures [7,13,16,17]. For some authors, it is the methodology of choice for the biomechanical analysis of implant-supported prostheses [16,17].

Although porcelains are widely used in oral rehabilitation [18] and have excellent esthetic resolution [18], resins seem to be more efficient than porcelains in reducing the transmission of impact forces due to their lower elasticity modulus [19,20,21]. Thus, some materials, such as PEEK and glass ceramics in resin matrix, have been developed as an alternative to ceramics to absorb masticatory loads and reduce load transmission to implants and peri-implant bone. The differential of this study is the use of a current combination of restorative materials: PMMA, glass ceramic over resin matrix, PEEK + lithium disilicate, metal–ceramic, lithium disilicate, zirconia + feldspathic and monolithic zirconia.

The resistance characteristics of polymeric and ceramic materials have been well described in the literature. From a biologic point of view, materials such as zirconia and PMMA in the form of nanoparticles have been studied due to their antimicrobial potential. In addition, recent studies reported that zirconia nanoparticles have antibacterial, anticancer and antioxidant properties, in addition to acting as biosensors and providing structural reinforcement [22,23].

Given this scenario, it is noteworthy that the intention of this work is to evaluate, by strain gauge analysis, the influence of the restorative material in the transmission of loads to the peri-implant alveolar bone using different restorative combinations that evenly simulate the biomimetic concept.

The null hypothesis is that the restorative material does not influence load transmission to the peri-implant bone.

## 2. Materials and Methods

In this experiment, a 90 × 45 × 32 mm polyurethane test base (Nacional Ossos, Jaú, Brazil) with two different densities was used. The first 2 mm simulated the density of a cortical bone (40PCF—0.96 g/cm^3^), and the remainder of the evidence base simulated a type III trabecular bone (15PCF—0.24 g/cm^3^). Polyurethane was therefore used because this substrate has been used in earlier works with strain gauge analysis to simulate human bone marrow. Polyurethane is a homogeneous and isotropic substrate [7].

**Figure 1:**
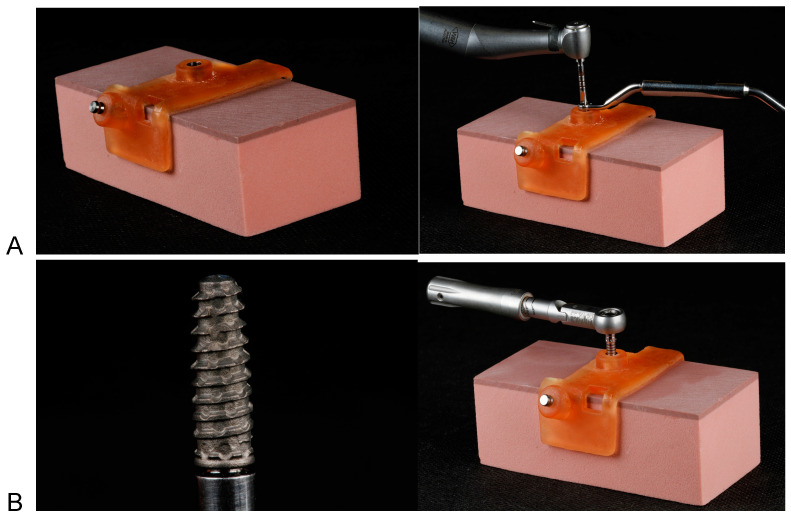

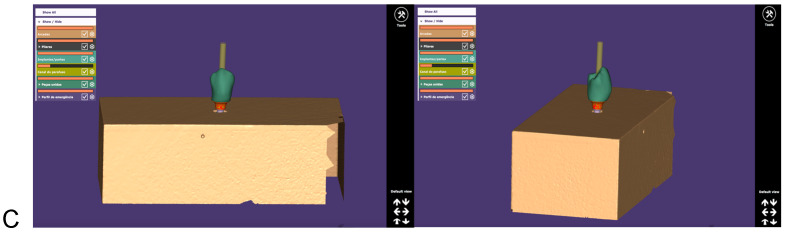
(**A**) Evidence base, surgical guide and drilling for implant installation. (**B**) Implant and installation with prosthetic ratchet. (**C**) Design of the tooth to be milled, carried out in Exocad.

In addition, a surgical guide was made, designed in 3Shape Implant Studio software, with the intention of allowing an installation perpendicular to the test base and 1 mm below the test base surface, providing implant fixation in a 1 mm cortical density. The guide was printed with a specific resin used in the manufacturing of surgical guides (Smart Print Bio Clear Guide) by means of a 3D printer (MiiCraft 125 Ultra) (Figure 1A).

The installed implant had dimensions of 3.5 × 11 mm and a morse taper prosthetic connection (Maestro, Implacil De Bortoli). The guided surgery kit from the same manufacturer was used for test base installation, following their milling recommendations. Furthermore, an implant motor and a 20:1 reduction counter-angle (NSK) were used at a speed of 900 rpm (Figure 1B). After installation in the block, the implant had an initial stability, measured by a torquemeter, of 45 N.

After installing the implant, a scan body (Implacil De Bortoli) was installed over it, and the test base was scanned (Ceramill Map 400). The file generated in STL was imported into Exocad software, and the design of a mandibular first premolar was made (Figure 1C).

It is also important to mention that all fixed single unit prostheses and infrastructures were designed in Ceramill Mind software so that all samples had exactly the same shape (Figure 1C).

**Figure 2:**
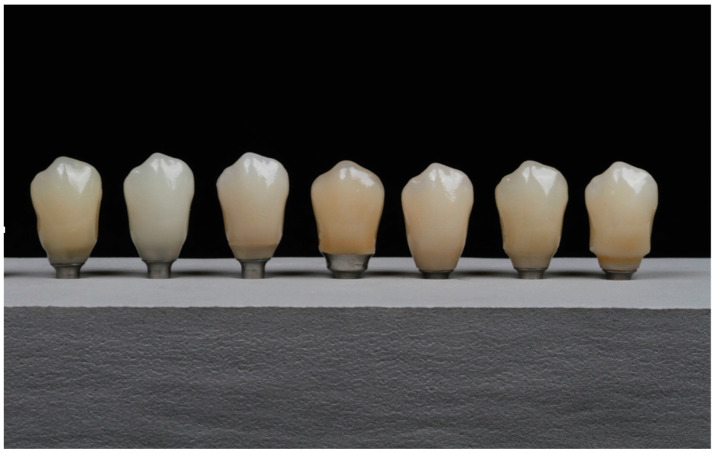
Similar crowns in shape and size made with different materials.

From this model, 7 similar crowns were made with 7 different material compositions, as shown in Figure 2. The crowns made using the CAD/CAM method were hybrid, that is, cemented on a titanium base, and the set was screwed onto the implant [21].

The crowns in PMMA, resin matrix ceramic, monolithic disilicate and monolithic zirconia were milled (Ceramill Motion 2) following the design project described above.

In the crown with PEEK infrastructure and lithium disilicate coating, the infrastructure design was reduced from the original design drawing, generating adequate space for milling the lithium disilicate coating according to the project described above and a crown with the same shape and dimensions as previously planned.

In the metal–ceramic crown, the infrastructure was cast using a machined wax infrastructure, and the feldspathic ceramic was layered following a silicon wall to copy the previously planned shape and dimensions.

In the crown with zirconia infrastructure and feldspathic ceramic coating, the infrastructure design was reduced from the original project design, generating adequate space for the ceramic layering and following a silicon wall to copy the previously planned format and dimensions.

All crowns were sandblasted with 50 micron aluminum oxide at a distance of 2 cm for 30 s, rinsed in running water for 1 min and dried with an air jet. Prior to cementation, the zirconia and lithium disilicate structures were silanized (Alloy Primer Kuraray) following all adhesion protocols recommended in the literature [22]. In addition, all fabricated crowns were cemented to the abutment (4.5 mm in diameter, 6 mm in height and 2.5 mm in transmucosal length—Implacil De Bortoli) with U-200 (3M) self-adhesive dual cement, respecting all manufacturer guidelines.

**Figure 3:**
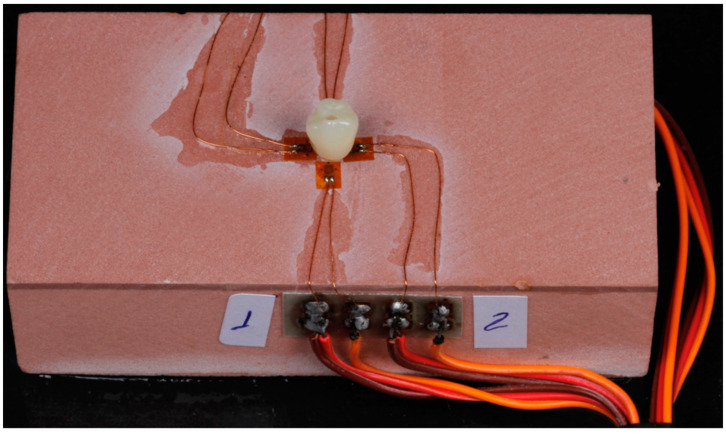
Position of the strain gauges pasted onto the test base. (1- vestibular 2- mesial).

Four linear strain gauges (Excel sensors) were installed in the test base around the implant, simulating mesial, distal, buccal and lingual positions. Furthermore, a small amount of cyanoacrylate (Super Bonder, Loctite) was also used for their fixation. After correct positioning, the sensors were subjected to light pressure, and the adhesive was allowed three minutes to dry (Figure 3).

It is also important to emphasize that the position of the strain gauges was chosen to simulate the buccal, lingual, mesial and distal positions, and they were all connected to the strain gauge conditioning unit as shown in Figure 3.

The strain gauges were connected to an electrical signal conditioning unit (Excel Sensors) using shielded electrical cables. Each strain gauge formed a connection called a 120-A Wheatstone Bridge, which is an electrical circuit that is suitable for detecting minute changes in electrical current caused by strain [7].

These variations occur on a millionth scale (microvolts) and are recorded and amplified by the signal conditioning unit, Strain Gauge Bridge Kit (model MDC-10; Transtec, São Paulo, Brazil; FAPESP 2012/50560-0), which, in addition to feeding the Wheatstone Bridges, amplifies the generated signal and performs the analog-to-digital conversion.

**Figure 4:**
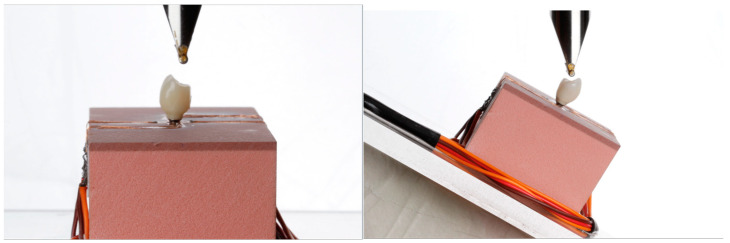
Tip of the universal testing machine (Kratos) inciting axially and obliquely at 45°.

The 7 different crowns were tested on the Universal Testing Machine (2000 RK Kratos) under 2 different conditions (Figure 4):

- Axial load, 150 N;
- 45° oblique load, 150 N.

The 7 crowns (Table 1) under the 2 test conditions (axial and 45°) generated 14 groups, and 5 measurements were performed in each group. The obtained measurements generated a total of 280 results (7 models, 4 strain gauges, 2 different conditions and 5 measurements each) and were statistically analyzed.

The magnitude of strain measured by each strain gauge was recorded in microstrain （με）. After each load application, there was a 3 min wait for elastic recovery of the polyurethane, and, before each reading, the device was balanced and calibrated with no tensions in the experimental model. All measurements were performed by a single operator.

The data obtained were tabulated, and intra- and inter-group analyses were performed. After confirming the normality of the data by the Shapiro–Wilk test, the ANOVA test with Tukey’s post hoc test was chosen to compare the 7 different materials and the Student’s *t*-test to compare the axial and 45-degree oblique loads. The level of significance was set at *p* ≤ 0.05.

## 3. Results

After confirming data normality using the Shapiro–Wilk test, an ANOVA with Tukey’s post hoc test was conducted to compare the seven different materials, followed by a Student’s *t*-test for comparison between the axial and 45-degree loads. The significance level was set at *p* ≤ 0.05. There was no statistically significant difference in the evaluation of the materials, both in the axial load and in the oblique load at 45 degrees.

**Figure 5:**
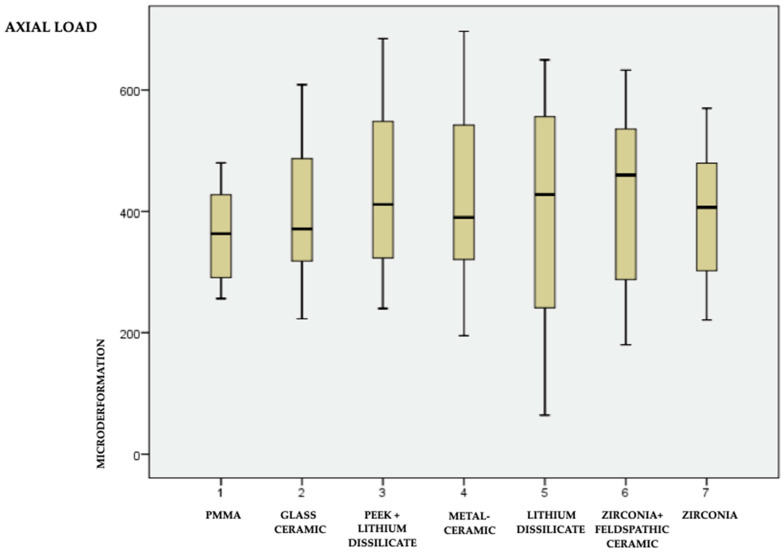
Means, minimum and maximum values of the 7 materials under axial load.

**Figure 6:**
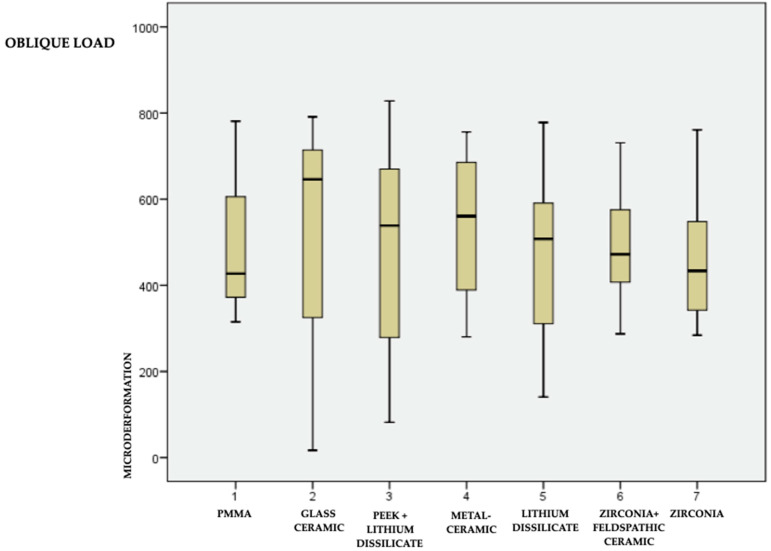
Means, minimum and maximum values of the 7 materials under 45° load.

The data are in Table 2 and graphically represented in box plot form (Figure 5 and Figure 6 for the axial and oblique loads, respectively). The representation of materials in Figure 5 and Figure 6 follows the arrangement of materials in Table 1.

Finally, in the individual comparison of materials between axial load and oblique load, we noticed a greater load transmitted to the bone when subjected to a 45° oblique load compared to an axial load.

## 4. Discussion

The clinical success and longevity of implant-supported treatments depend on a series of factors. In addition to biological factors such as bone quantity, implant position and size and peri-implant soft tissue quality, technical aspects are also very important in determining long-term clinical success and include the choice and combination of materials, the fabrication method and the space available beyond the occlusion’s biomechanical control [24,25,26]. Overload on implants is related to mechanical complications or treatment failure after placing the implants in function [2]. These mechanical complications can manifest in implants, implant-supported prostheses or supporting bone tissue, and the most commonly reported are: screw loosening or fracture, fracture of occlusal covering/coating materials, fracture of the prosthesis, crestal bone resorption and fracture with consequent loss of implants [27].

Studies have demonstrated that several factors influence peri-implant bone loss, from the implant platform to the periodontal biotype, the moment the implant is installed and occlusal overload [8].

This study used an implant with a morse cone connection, which is potentially more stable, has less bacterial infiltration and clinically generates less bone loss [28], to assess the influence of the restorative material in this process of transmission of masticatory loads to the peri-implant bone. Finite element analysis has shown a better load distribution to the alveolar bone in morse taper implants when compared to other types of prosthetic connection [29], so this may have been one of the factors responsible for the similar results of load transmission to the bone in this research regardless of the materials chosen for this study.

In this case, a narrow 3.5 mm diameter implant was used. In this regard, recent systematic reviews that evaluated clinical success with narrow implants (3.3 and 3.5 mm in diameter) showed that there is no difference between these and regular implants, both for marginal bone loss and for longevity [30,31,32].

It is also important to highlight that a test base in polyurethane was used in this study to follow the standards of ASTM F1839-08.25 for the manufacture of the experimental model. Polyurethane was used in previous works with similar methodologies to simulate human bone as it has a bone-like elasticity modulus, in addition to being a homogeneous and isotropic substrate [7,16,33,34,35,36].

In a finite element analysis study evaluating the influence of different types of cement on stress distribution in monolithic restorations, it was concluded that resin cements better distribute stresses and therefore were used in the link/crown interface for the fabrication of implant-supported, screw-retained crowns [37].

It was also observed that several previous studies used biomechanical methodologies simulating masticatory loads, opting for loads between 100 N and 150 N in their methodologies, which the authors replicated in this study [7,38,39].

The elasticity modulus is the resistance of an object or substance to elastic deformation (i.e., non-permanent deformation) when a force is applied to it. Lithium and zirconia have high strength and a low elasticity modulus, which makes them friable without deformation capacity. Materials with a lower modulus of elasticity, such as, for example, resin-based materials PMMA (polymethylmethacrylate), PEEK (polyether ether ketone) and hybrid resins, can be a viable alternative, as they deform and thus reduce the transmission of forces [40,41,42,43].

Hybrid abutment crowns are used because they allow customization of the subgingival contour in addition to the use of different types of materials with the same shape. This is a solution that allows the cementation of different materials on a metallic link, and the set can, in this way, be screwed onto the implant. Edelhof et al. described the advantages of a simple fabrication process and the possibility of extra-oral cementation and the disadvantages of risk of loss of retention due to cementation failures and the lack of long-term evidence [24,44].

Currently, several types of hybrid resins are available, including resin nanoceramics (Lava Ultimate; 3M ESPE, Cerasmart; GC Corp), and polymer-infiltrated hybrid ceramic (Vita Enamic; Vita Zahnfabrik) [45] materials have been developed to resist chipping and fracture, as well as to absorb masticatory forces [46].

Polyether ether ketone (PEEK) is a synthetic thermoplastic polymer that has high mechanical performance and has been used as a biomaterial in medical applications. Recently, attention has been paid to the use of PEEK in place of metal alloys as an implant material, coating material, CAD–CAM milled frame material and abutment material. In addition, within the biomimetic concept, the compatibility between the PEEK biomaterial’s modulus of elasticity and bone can be used as an infrastructure under a ceramic covering to reduce load transmission to the peripheral bone [47].

Complications such as fractures and chipping were commonly found in the literature when using two-layer [48] restorations and zirconia as infrastructure material. Thus, mainly due to this fact, monolithic materials are being developed.

In this scenario, no statistically significant difference was observed in the transmission of load to the bone with the seven different materials. Junior et al. [49], in a finite element biomechanical assessment, found similar results, in which the restorative material also did not influence the load transmitted to the bone.

Likewise, in a systematic review, Abou-Ayash et al. [50] stated that current evidence suggests the prosthetic material selection has no influence on the medium- and long-term survival of implants restored with single crowns and fixed partial dentures. The following materials were evaluated in this clinical study: metal–ceramic, lithium disilicate, veneered zirconia, veneered alumina and nanoceramics. Likewise, prosthetic material appears to have no significant impact on prosthetic survival rates.

In another similar study, comparing different configurations of hybrid crowns and using a photoelastic analysis methodology, Abarno et al. found different results and showed that crowns in zirconia have the highest transmission of forces when compared to feldspathic ceramics and lithium disilicate [15].

Corroborating this study, Wang et al. [51], using FEA, stated that the total energy transferred to the bone–implant interface is similar regardless of the restorative material, although restorative crowns made of different materials may have different impact absorptions.

Based on the results, there was no difference between monolithic ceramic restorations, whether disilicate or zirconia, and restorations made of polymeric materials, in the same way that there was no difference between two-layer restorations, whether metal–ceramic or within the biomimetic concept of PEEK infrastructure and ceramic cover. That is, the restorative material did not influence the load transmission to the supporting bone. Furthermore, although differences between the axial and oblique loads were evident, all materials behaved similarly for each type of load.

The results of the current study are strongly related to and in line with a recently published clinical study [52]. This study evaluated the effect of implant-supported fixed porcelain-to-metal dentures and indirect composite resin on peri-implant marginal bone resorption, and no difference was found at 18-month follow up.

As limitations of this study, we can report that only one single load (150 N) was used. Strain gauge analysis only measures deformation at the bone crest and does not allow the assessment of load dissipation along the bone as in a photoelastic analysis. Some differences in the shape and morphology of the crowns, especially those that received the manual addition of ceramic, and the use of only one sample of each type of crown can be considered limitations.

It is noteworthy that this was an in vitro biomechanical study, therefore, it could not reproduce clinical conditions, such as the patient’s bone density, bone implant contact or type of occlusion, among other factors. Therefore, further clinical studies must be performed to confirm the results obtained in this study.

## 5. Conclusions

Despite the limitations of this study, the axial and oblique load transmissions to the peri-implant bone crest were not influenced by the different modulus of elasticity of the seven different types of material sets, whether polymeric or ceramic, monolithic or double layer. The choice of the restorative material, therefore, should be based on other parameters such as esthetics, type of antagonist, occlusal pattern and longevity and not on the modulus of elasticity of the material in question.

## Figures and Tables

**Table 1:** Description of the 7 materials used and their manufacturers.

1	PMMA—polymethylmethacrylate (VIPI Block Trilux)
2	Glass ceramic in resin matrix (GC—Cerasmart)
3	PEEK—thermoplastic polymer (Juvora) with lithium disilicate coating (Rosetta)
4	Metal–ceramic (NiCr + GC Initial—MC Classic)
5	Lithium disilicate (Rosetta)
6	Zirconia infrastructure (Zolid Amann Girrbach) and feldspathic ceramic coating (Leucite GC Initial LRF)
7	Monolithic zirconia (Zolid Amann Girrbach)

**Table 2:** Averages in microdeformation (με) and standard deviation of the 7 different materials for axial and oblique loads.

Materials	Axial Means	Axial Deviations	45° Mean	45° Deviation	*p*-Value
1—PMMA	361.70	75.487	486.00	151.392	0.00
2—Hybrid resin	402.45	112.629	515.40	282.465	0.20
3—PEEK + disilicate	437.55	149.387	487.25	250.764	0.15
4—Metal–ceramic	427.80	148.991	533.35	166.678	0.00
5—Monolithic disilicate	391.80	200.403	471.95	201.646	0.00
6—Zirconia + disilicate	418.80	143.448	496.30	127.304	0.00
7—Monolithic disilicate	397.60	114.196	462.75	139.496	0.00

## Data Availability

Publicly available datasets were analyzed in this study. This data can be found here: [https://www.dropbox.com/sh/6dz2xv7k8u5f8x8/AABT-1ajGmgCakX47PQUlaoqa?dl=0].
